# The Revolutionary Roads to Study Cell–Cell Interactions in 3D In Vitro Pancreatic Cancer Models

**DOI:** 10.3390/cancers13040930

**Published:** 2021-02-23

**Authors:** Donatella Delle Cave, Riccardo Rizzo, Bruno Sainz, Giuseppe Gigli, Loretta L. del Mercato, Enza Lonardo

**Affiliations:** 1 Institute of Genetics and Biophysics “A. Buzzati-Traverso”, National Research Council (CNR-IGB), Via Pietro Castellino 111, 80131 Naples, Italy; donatella.dellecave@igb.cnr.it; 2 Institute of Nanotechnology, National Research Council (CNR-NANOTEC), c/o Campus Ecotekne, via Monteroni, 73100 Lecce, Italy; riccardo.rizzo@nanotec.cnr.it (R.R.); giuseppe.gigli@unisalento.it (G.G.); loretta.delmercato@nanotec.cnr.it (L.L.d.M.); 3 Department of Cancer Biology, Instituto de Investigaciones Biomedicas “Alberto Sols” (IIBM), CSIC-UAM, 28029 Madrid, Spain;bsainz@iib.uam.es; 4 Spain and Chronic Diseases and Cancer, Area 3-Instituto Ramon y Cajal de Investigacion Sanitaria (IRYCIS), 28029 Madrid, Spain; 5 Department of Mathematics and Physics “Ennio De Giorgi”, University of Salento, via Arnesano, 73100 Lecce, Italy

**Keywords:** pancreatic cancer, 3D cultures, tumor microenvironment, fluorescent microscopy, nanotechnology, predictive models

## Abstract

**Simple Summary:**

Pancreatic cancer is an extremely lethal malignancy with a survival rate lower than any other cancer type. For decades, two-dimensional (2D) cultures have been the cornerstone for studying cancer cell biology and drug testing, due to their simplicity and cost. However, their inability to reconstitute the tumor architecture, the absence of nutrient and oxygen supply gradients, as well as the lack of appropriate mechano-forces that mimic the extracellular microenvironment, make them an inadequate model to accurately reproduce tissue level-specific characteristics. Bioengineering systems, such as three-dimensional (3D) patient-specific models, are progressively emerging as systems better able to mimic the biology of pancreatic tumors and to test new anticancer therapies, as they more efficiently recapitulate the complex tumor microenvironment characteristic of pancreatic tumors. Here, we review how cellular component interactions, within the pancreatic tumor microenvironment, have been studied and mimicked in 3D cell culture models, and discuss selected emerging therapeutic strategies, addressing their limitations and future perspectives.

**Abstract:**

Pancreatic cancer, the fourth most common cancer worldwide, shows a highly unsuccessful therapeutic response. In the last 10 years, neither important advancements nor new therapeutic strategies have significantly impacted patient survival, highlighting the need to pursue new avenues for drug development discovery and design. Advanced cellular models, resembling as much as possible the original in vivo tumor environment, may be more successful in predicting the efficacy of future anti-cancer candidates in clinical trials. In this review, we discuss novel bioengineered platforms for anticancer drug discovery in pancreatic cancer, from traditional two-dimensional models to innovative three-dimensional ones.

## 1. Introduction

Pancreatic cancer (PC) is a devastating and essentially incurable disease, leading to patient death in the majority of cases [1]. Incidence and mortality are increasing steadily, and PC is predicted to become the second leading cause of cancer-related death by 2030 [2]. According to the Italian Association of Medical Oncology (AIOM), in 2019, approximately 13,500 new PC cases were diagnosed in Italy [3]. These dramatic numbers are due largely to late diagnosis, lack of effective therapies, and a poor understanding of PC biology. Thus, PC has become a world healthcare priority, and studies focused on novel in vitro models to investigate tumor progression and develop more effective treatments are urgently needed. One reason for the lack of success for the majority of drugs used to treat PC patients is their inappropriate purposing and associated toxicity. At the preclinical stage, two-dimensional (2D) cultures have been a milestone in the study of cancer biology, since they represent a useful platform for analyzing genetic and molecular alterations and a cost-effective system for drug screening [4]. However, they are an inherently and extremely simplified model that fail to precisely reflect the human tumor microenvironment (TME) and its molecular components [5], which can inevitably lead to non-translatable results [6,7]. Those aspects are crucial for the study of PC, characterized by an abundant desmoplastic core, which accounts for up to 90% of the total tumor volume, and by an intricate cross-talk among tumor and stromal cell, both of which are critical aspects for cancer progression [8,9]. The TME is a very complex ecosystem in which several cellular (such as pancreatic stellate cells (PSCs), cancer-associated fibroblasts (CAFs), and immune cells) and non-cellular components (such as cytokines, immunoregulatory molecules, and extracellular matrix (ECM) components) are involved, contributing to the development of a hypoxic and “cold” immunosuppressive tumor, resistant to chemotherapy, targeted therapy, and immunotherapy [10]. While the number of drugs targeting the TME is progressively increasing, there is an urgent demand for more biologically and physiologically relevant in vitro preclinical models that can accurately mimic the TME that exists in patient tumors. The limitations of 2D models have been, in part, overcome by the use of genetically engineered mouse models (GEMMs), which recreate the most frequent genetic alterations associated with pancreatic cancer progression and provide a more physiological microenvironment for drug testing and genetic research [11]. However, in vivo studies are expensive, time-consuming, and ethically not suitable for drug screening and, most important, the results obtained have limited relevance in humans [12]. The opportunity of reproducing the TME in vitro by growing tumor cells in three-dimensional (3D) matrixes or scaffolds has opened new horizons in the field of drug testing. These 3D in vitro tumor models have been shown to be superior to 2D models, allowing for multiple cell populations to interact and mimicking the complexity and the biomechanical properties of tumors as well as tumor heterogeneity, and allowing for treatment responses similar to those seen in patients diagnosed with PC [13,14,15,16,17].

## 2. 3D In Vitro Models

3D in vitro systems are advantageous predictive tools, which may accelerate translating basic research into personalized medicine by providing more physiological information on cellular responses to different stimuli [18]. 

### 2.1. Spheroid 

**Figure 1:**
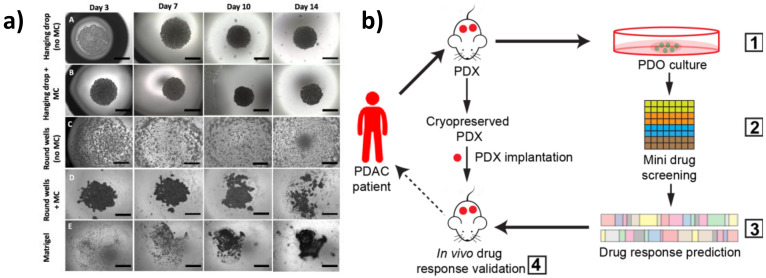
Three-dimensional (3D) in vitro models of pancreatic cancer. (**a**) MiaPaCa-2 spheroid growth using different methods. Scale bars = 1000 μm (adapted from [21]). **(b**) Schematic representation of the patient-derived organoid (PDO)-derived system. (1) Generation of PDO and patient-derived xenograft (PDX) models from cryopreserved xenografts of patients with pancreatic ductal adenocarcinoma (PDAC). Screening of Food and Drug Administration (FDA)-approved drugs (2) and validation in organoid cultures (3). Selection of effective drugs for small-scale drug screenings and validation in the PDXs (4) (adapted from [30]).

Spheroids are the simplest 3D model, which can be generated by the hanging drop technique, consisting of embedding cells in different matrices (such as collagen, methylcellulose, gelatin, and alginate hydrogels), or by growing single-cell suspensions in ultra-low attachment plates, in order to minimize the adhesion to plastic supports and to optimize cell–cell and cell–matrix interactions [19]. Spheroids can arise from self-assembling cells or via cellular aggregation and constitute an easy and highly reproducible 3D model [20]. Recently, Cavo and colleagues developed a new method to generate spheroids from the pancreatic ductal adenocarcinoma (PDAC) cell line (i.e., MiaPaCa2) with the hanging drop technique. They used a methylcellulose-enriched media and hydrophobic substrates to generate well-organized pancreatic spheroids with increased mesenchymal and cancer stem cell features [21]. This method appears to be efficient for the long-term generation of spheroids, even from those cell lines that hardly give rise to 3D structures due to low cohesiveness (Figure 1a). By comparing 2D cultures with 3D spheroids generated by different techniques (e.g., ultra-low adhesion concave microwells, Matrigel inclusion, and organotypic systems), Zeeberg and colleagues found differences in cell growth, morphology, and in the response to different stimuli. They concluded that the organotypic culture, generated by plating cells on the top of a matrix gel, more closely recapitulated the tissue architecture of PC [22]. 

### 2.2. Patient-Derived Organoids (PDOs)

PDOs are 3D structures established from freshly isolated primary cells, which retain the ability of self-renewal and self-organization in an organ-like structure that recapitulates the tissue of origin. A pioneer study in establishing pancreatic organoids from both normal and freshly isolated human tumor biopsies was performed by Boj et al. [23]. The authors optimized the standard culture condition protocols for generating organoids able to recapitulate disease-specific alterations, enabling them to recreate in vitro the different stages of tumor progression. Specifically, single-cell suspensions are embedded in a Matrigel or collagen matrix and supplemented with a media containing a well-defined combination of tissue-specific growth factors [23,24,25]. PDOs can be genetically engineered in vitro by CRISPR-Cas9 (Clustered Regularly Interspaced Short Palindromic Repeats) technology to specifically edit tumor-driving genes, and can be xenografted in immunodeficient mice for in vivo studies [26]. 

The potential of pancreatic PDOs has also been recently exploited for the development of a PDO-based gene expression signature [27] and for the establishment of PDO biobanks [28]. PDOs represent a powerful model to recapitulate tumor histology and for personalized drug screening. Intriguingly, Seino and colleagues identified three functional PDO subtypes in PDAC on the basis of differences in stem cell niche factor dependency. By engineering them with clustered regularly interspaced short palindromic repeats (CRISPR)-Cas9 technology, they found that exogenous WNT is required to support the initial stages of tumorigenesis but it is dispensable in the late stage of tumor progression [29]. Pancreatic PDOs have also been shown to be as effective at determining drug efficacy compared to more labor-intensive and cost-prohibitive in vivo patient-derived xenograft (PDX) models. For example, Frappart et al. published a small-scale drug screening PDO platform that was successfully validated in an in vivo xenograft trial, highlighting the clinical utility of PDOs for validating and discovering new treatments for PC [30] (Figure 1b). Moreover, Nelson et al. used different multidisciplinary approaches, which included confocal microscopy and transcription profiling analysis, to compare PDX organoids with isogenetically matched 2D primary cell lines from PDAC tumors and primary cell line organoids (CLOs) [31]. Interestingly, they developed an in vitro method to generate CLOs, which very closely recapitulated the main features of organoids generated from PDXs, thus overcoming the limitations associated with xenografts. Along these lines, PDO drug screening platforms have recently been improved by the development of automated organoid-based platforms that take advantage of microfluidic systems for the simultaneous and time-controlled test of thousands of compounds, enabling real-time genetic and phenotypic analysis of PDOs, serving as a step forward in personalized therapy [32].

## 3. Reconstitution of Tumor Cell Heterogeneity and Complexity in 3D In Vitro Models

PC is characterized by a dense desmoplastic reaction defined by different cellular components, such as immune cells, fibroblasts, endothelial cells, and PSCs, dispersed in an organized extracellular matrix enriched in collagens, hyaluronic acid, and laminins [33,34]. The TME is therefore conditioned by distinct environmental factors, i.e., cytokines, growth factors, as well as a specific biochemical profile, which mediate cellular communication and are crucial in influencing cancer progression [35,36]. Reconstituting the tumor complexity by using 3D models requires taking into consideration the specific interactions between matrix components and the different cellular types present in the tumor in order to recreate in vitro the specific environmental conditions that distinguish each tumor. Attempts to recreate such a complex environment is also one of the ways to master in vitro tumor modeling. 

### 3.1. Scaffolds

An optimal scaffold should offer a suitable environment for cell growth and allow for the development of in vitro tumor models that thoroughly recapitulate cell–extracellular matrix exchanges. In this scenario, tumor cells can be cultivated within biomaterials, including de-cellularized native tissues or in 3D synthetic and/or natural scaffolds. The biophysical properties of 3D structures are critical aspects for the study of PCs, as demonstrated by Puls et al., who generated a 3D PC model by embedding PDAC cells into different matrices obtained from type I collagen and Matrigel at various percentages. The authors found that matrix stiffness causes epithelial to mesenchymal transition (EMT) and induces the growth of cells as tight clusters [37]. Chiellini et al. developed a 3D model in which PDAC cells were embedded in a hydrogel made of chitosan (mCS) or a polyelectrolyte complex (mPEC) between CS and poly(g-glutamic acid) (g-PGA). These systems allowed for the generation of spheroids with enhanced features associated with cancer invasiveness [38]. Ricci et al. generated scaffolds by combining different polymeric formulations and different techniques in an attempt to reproduce various structures and ECM features, as well as to test their influence on tumor growth. They found that spongy scaffolds obtained via emulsion-based and salt leaching-based techniques increased cell viability and aggressiveness by inducing a spatial organization of tumor cells that closely recapitulated those found in vivo [39]. The advancements in the field of tissue engineering (TE) have facilitated the use of scaffold-based 3D culture systems in which cells are induced to colonize rigid micro- or macro-porous structures made of natural or synthetic fibers that mimic the ECM architecture [40]. Scaffold can be synthesized by modulating different parameters, such as stiffness and porosity, in order to sustain cell viability and to mimic the morphology of the original tissue [41,42]. To better reproduce the TME architecture, Totti et al. developed a porous polyurethane scaffold coated with fibronectin, one of the main components of the extracellular matrix, and they showed that this system enhanced long-term tumor proliferation and collagen-I production compared to uncoated scaffolds, as well as induced a spatial oxygen and nutrient gradient [42]. 

### 3.2. 3D Cell/TME

**Figure 2:**
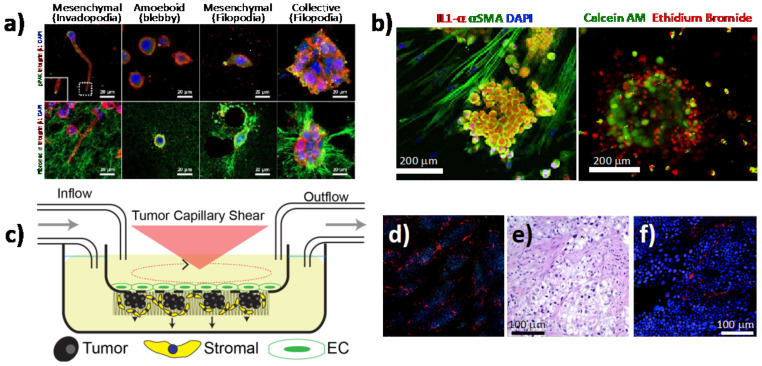
Heterotypic 3D in vitro models of pancreatic cancer. (**a**) Plasticity and mechanoreciprocity in pancreatic cancer (PC) cell migration and cell–extracellular matrix (ECM) interactions. Cancer cells showed individual or collective cell migration in 3D ECM environments. Individual PANC-1 cells formed actin-rich protrusions on the plasma membrane (invadopodia). PANC-1 cells showed organized ECM fibers along the direction of invadopodium growth. Individual BxPC-3 cells appeared as either a rounded shape (amoeboid) without podium formation or as a mesenchymal shape with actin-spike protrusions (filopodia). The mechanoreciprocity of cell–ECM interactions appeared to be associated with integrin-based adhesion. BxPC-3 cells showed extensive ECM deformation and unfolding around the filopodia along with a FAK-mediated traction force (adapted from [51]). (**b**) Photodestruction of fibroblasts correlates with enhanced tumor response to photodynamic therapy (PDT) using verteporfin (adapted from [53]). (**c**) Schematic of 3D in vitro tumor microenvironment system. Endothelial cells are plated above the transwell, and pancreatic stellate cells (PSCs) and PDAC cells are plated below the transwell. Tumor-derived hemodynamic force is applied above the transwell to the endothelial cells through rotation of the cone. The upper and lower chambers are independently perfused with media to recapitulate interstitial flow. (**d**) Immunofluorescence of PDAC cells (green, anti-cytokeratin 18) and PCSs (red, anti-fibroblast), nuclei stained with 4′,6-diamidino-2-phenylindole DAPI. 4x composite image. (**e**) Immunohistochemistry (IHC) of a PDAC clinical sample. (**f**) Immunofluorescence stained as in the left panel (adapted from [54]).

Although 3D cultures have significantly improved the analysis of cancer progression and drug response, compared to 2D cultures, they are still far from recapitulating the complexity of the TME in vitro. The heterotypic 3D tumor culture, obtained by co-culturing different cell types, provides a suitable system that reflects the physiological properties of PDAC tumors and a more realistic response to chemotherapeutic treatment [43,44]. As mentioned above, PDAC is characterized by a dense and fibrotic stroma, mainly composed by PSCs, which are quiescent cells that upon activation secrete abundant ECM proteins such as fibronectin and collagen [45]. The involvement of the stroma in PDAC chemo-resistance has been widely debated and researched [46]. It has been demonstrated to act as a supportive niche for PDAC cells, promoting their aggressiveness [47] and blocking chemotherapeutic drugs from reaching the tumor [34]. However, depletion of the stroma by the sonic hedgehog inhibitor (i.e., saridegib) in clinical trials has proven to produce more aggressive tumors and lower survival, which was also confirmed by three independent animal studies) [48,49]. A deeper understanding of the stroma and its crosstalk with the tumor cells is urgently needed. Towards this end, Norberg and colleagues developed a simple spheroid assay by co-culturing tumor and PSCs, both from human and mouse PDAC samples, in methylcellulose, followed by a “virtual sorting” to analyze the cross-talk between these two populations by using species-specific primers [50]. They found that the presence of stromal cells induced a mesenchymal phenotype and stimulated the proliferation of PDAC cells. In a different approach, Kim et al. co-cultured, in a microchannel device, PSCs and tumor spheroids generated from two different PDAC cell lines (with a mesenchymal and an epithelial phenotype, respectively) embedded in a collagen matrix. They observed differences in cell plasticity, extracellular matrix remodeling and migratory capacities on the basis of the PDAC cell types used for the co-culture [51] (Figure 2a). Broekgaarden et al. demonstrated that patient-derived CAFs increase PDAC resistance to oxaliplatin and benzoporphyrin derivative-mediated photodynamic treatment (PDT), both in vitro and in vivo, through the regulation of redox status [52]. This could be a useful tool for screening new drugs that modulate metabolic pathways. Karimnia et al. created a 3D model where PSCs were embedded in Matrigel that was overlaid with a single-cell suspension of PDAC cells, and they used this system to test oxaliplatin and PDT treatment [53]. They found that the PDAC cells were more resistant to chemotherapy and more sensitive to PDT treatments when co-cultured with fibroblast. This system allowed for a more physiological response assessment to drug treatments compared to homotypic cultures, suggesting that the PDT treatment could be used as a powerful approach for stroma depletion (Figure 2b) [53]. The lack of vasculature in the 3D models has been overcome by the generation of a multi-cellular 3D system, which combines tumor and stromal cells with microvascular endothelial cells exposed to shear stress and flow rates (Figure 2c–f). Transcriptome analyses revealed how this system closely mimics the true biology of the tumor [54]. In a 3D organotypic culture of endothelial, tumor, and stromal cells, Di Maggio et al. founded that PDAC, as well as a collagen-rich gel, suppressed the pro-angiogenic role of PSCs on endothelial cells, thus inhibiting their proliferation. This appears to be a valid model for testing the effect of anticancer drugs on tumor vascularity [55]. The tri-culture system was optimized by Gupta et al., who established a polyurethane (PU) scaffold-based model in which tumor cells were seeded in an inner compartment coated with fibronectin, surrounded by an external collagen-coated compartment containing stromal cells. This system allowed for the activation of PSCs and the trans-migration between the two compartments [56]. Fook Lun Lai et al. generated a 3D vascularized network by engineering a co-culture, consisting of human PDOs, fibroblasts, and endothelial cells, on a perfusable platform that allows for the study of cell metabolic activity and drug screening [57]. PDAC consists of an extremely immunosuppressive microenvironment, and in order to study its contribution to chemotherapy resistance, Tsai and colleagues developed a multi-cellular organotypic model with stroma, tumor, and immune cells, which mimicked authentic TME interactions and was shown to be suitable for translational medicine in vitro [58]. Kuen et al. demonstrated the in vitro differentiation of monocytes into M2-like macrophages when grown in a 3D spheroid model, which included PDAC cells and fibroblasts [59]. This model could be used to analyze the tumor–stroma crosstalk influenced by the immune system. Finally, Ohlund and colleagues set up a 3D co-culture system of CAFs and tumor cells. The authors found two distinct subtypes of CAFs, one located closer to the cancer cells, which exhibited inflammatory and immune-suppression features, and another one more distant, with a myofibroblast-like phenotype supporting tumor growth. This work changes the classical view of the TME, proposing a more precise therapy to eradicate only a specific sub-population of stromal cells [10]. 

### 3.3. 3D suspension Bioreactors

**Figure 3:**
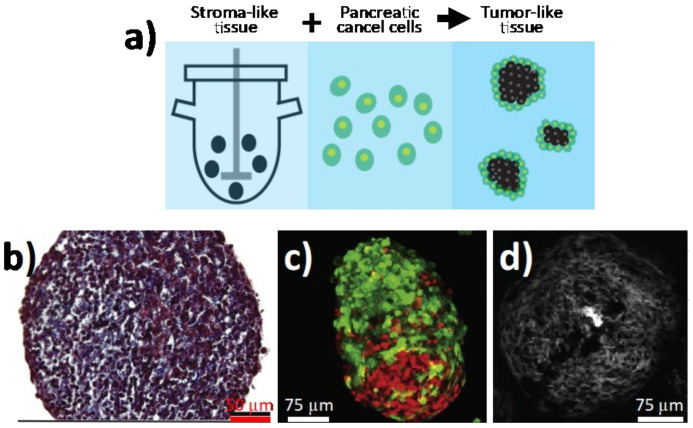
Bioengineered tumoral microtissues recapitulate desmoplastic reaction. (**a**) Schematic representation of the approach used for developing 3D in vitro PDAC bioreactor models. Stromal microtissues are produced by co-culturing normal fibroblasts or cancer-associated fibroblasts (CAFs) with gelatin microscaffold in a spinner bioreactor. After 6 days, PC cells (PT45) are added and the culture is stopped at day 12 for collecting PDAC microtissues in order to perform further investigations. (**b**) Masson’s trichrome staining and (**c**) confocal imaging for CAF/PT45 microtissues at day 12 of culture. (**d**) Second harmonic generation signal (gray scale) from newly formed fibrillar collagen in CAF/PT45 microtissues (adapted from [62]).

While not as common as the above-described 3D spheroid cultures or PDOs, 3D suspension bioreactor culture systems represent alternative models that may better mimic the unique physiological conditions of tumors (e.g., shear forces and addition of multiple cell types). For example, bioreactors can overcome the de-differentiation and loss of specialized cellular functions, which occurs when cells are removed from their host tissue and grown as monolayers. Several bioreactor platforms exist, including the rotating wall vessel (RWV), bioreactor cartridges, or spinner flask bioreactors, each presenting unique advantages. For example, RWVs offer an optimized suspension culture by providing a low-shear, low-turbulence environment that minimizes mechanical cell damage [60], as its horizontally rotating cylindrical vessel reduces shear and turbulence associated with conventional stirred bioreactors [61]. Nonetheless, RWVs have not been as widely used for PC compared to other bioreactor platforms. For example, Brancato and co-workers applied a tissue engineering approach to produce human PDAC microtissues by co-culturing PC cells (PT45) and normal fibroblasts or CAFs within biodegradable microcarriers in a spinner flask bioreactor (Figure 3a) [62]. Morphological and histological analyses showed that the presence of fibroblasts resulted in the deposition of a stromal matrix rich in collagen, leading to the formation of tumor microtissues composed of a heterotypic cell population embedded in their own ECM (Figure 3b-d). In a different approach, Kirstein et al. developed a bioreactor cartridge-based 3D PC cell system with the ability to infuse chemotherapies at concentrations similar to those found in biological samples, such as human plasma. The authors showed that this platform was useful for assessing the role of drug pharmacokinetics and delivery optimization of anticancer treatments, such as gemcitabine [63]. Similarly, Candini et al. recently described a flat, handheld, and versatile 3D cell culture bioreactor in which the PC cell line BxPC3 could be cultured, monitored in real time, and used in 3D cytoxicity assays [64]. The PetakaG3 LOT cell culture bioreactors, which are self-regulated, self-contained cell culture bioreactors that control oxygen, CO_2_, pH, and evaporation, have also been tested for delivery of chemotherapeutics to PC cells under conditions of hypoxia [65]. While bioreactors undoubtedly possess unique advantages, they have yet to become common alternative in vitro models for PC drug discovery.

## 4. Investigating Cell–Cell Interactions in In Vitro 3D Models

### 4.1. 3D Bioprinting

The use of scaffolds to generate 3D models has opened the way for innovative techniques such as 3D bioprinting. Cell printing combines scaffolds and different cell types to create a complex model with precise structure and high reproducibility [66]. Bioprinting facilitates a controlled spatial and temporal distribution of cells [67]. A 3D bioprinted tumor model can be generated by different techniques: inkjet printing, extrusion-based printing (EBP), laser-assisted bioprinting (LAB), and stereolithography [68]. 3D bioprinting has recently been used by Langer et al. to form multicellular structures consisting first of a PDAC cell core surrounded by primary human PSCs and umbilical vein endothelial cells (HUVECs). The authors demonstrated that multi-cell-type bioprinted tissues can recapitulate aspects of the in vivo tumor and provide a tunable system for the examination of several tumorigenic endpoints in the distinct tumor microenvironments [68]. Hakobyan et al. established a spheroid-based array using 3D bioprinting technology capable of reproducing the different stages of PDAC development in order to improve the understanding of PC tumor biology. This model allowed them to induce in vitro acinar to ductal cell transdifferentiation, a crucial process in PDAC progression [69].

Nevertheless, one of the major limitations of these 3D models is the lack of vasculature, which not only provides a supply of oxygen and nutrients but is essential for cancer metastasis. To overcome this limitation, microfluidics has emerged as a cutting-edge technology for combining the cellular reproducibility of PDOs with the flow control of a tumor-on-a-chip platform [57]. 

### 4.2. Organ-on-A-Chip

**Figure 4:**
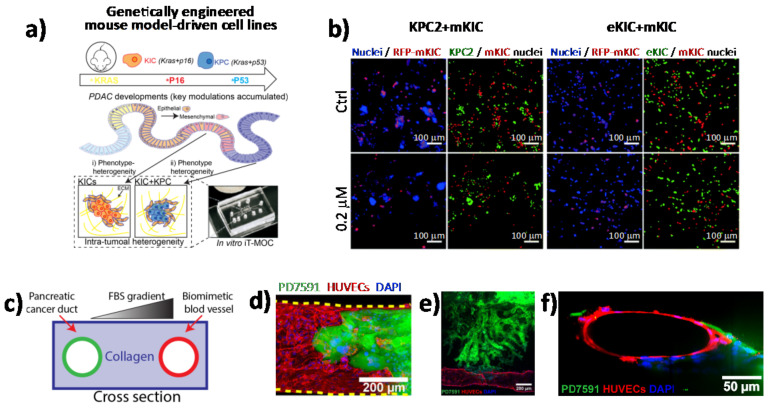
Direct imaging of cell–cell interactions in 3D in vitro models of PC. (**a**) Schematic illustration of the functional model of the in vitro intra-tumoral heterogeneous features composed by genetically engineered mouse model-driven cell lines to capture different PDAC progression stages. (**b**) Representative fluorescent micrographs of co-cultured KPC2–mKIC and eKIC–mKIC in control (Ctrl), 0.2 μM, and 20 μM gemcitabine treatment groups. Nuclei (blue) of each cell are distinguished in green (KPC2 and eKIC) and red (mKIC). Abbreviations: iT-MOC (interstitial tumor-microenvironment-on-a-chip), KIC genotypes (Kras mutation and Cdkn2a deletion), mKIC (mesenchymal phenotype KIC), murine pancreatic cancer cell lines (KPC2, eKIC, and mKIC). (a,b) (adapted from [77]). (**c**) Schematic illustration of PDAC-on-a-chip with a biomimetic blood vessel and a PC duct. One channel is seeded with endothelial cells to form a perfusable biomimetic blood vessel, while the other channel is seeded with PC cells to form a tumor duct. FBS (fetal bovine serum). (**d**) Representative confocal image of a section of the blood vessel (in red) invaded by YFP PD7591 PDAC cell (in green), showing that part of the blood vessel is being ablated by cancer cells in the organotypic model. (**e**) YFP PD7591 cells (in green) invading the biomimetic blood vessel (in red), migrating along the vessel and wrapping around the blood vessel. (**f**) Cross-sectional image of the biomimetic blood vessel shown in (**e**). (c-e) (adapted from [79]).

Although it is clear that tumors are heterogeneous mixtures of cells and ECM components, the extent to which different cell types influence cell–cell interactions as well as the paracrine signaling that is generated during therapy remains poorly understood. Nowadays, numerous microscopy-based imaging techniques are available to analyze cell morphology and cell–cell interactions within in vitro tumor models, including confocal microscopy, two-photon microscopy, and light sheet fluorescence microscopy [70,71,72]. One of the main advantages of using microscopy-based imaging approaches is the ability to observe spatial relations among different cell types with high temporal resolution under physiological conditions [73]. In this scenario, in vitro PDAC-on-a-chip provides powerful platforms to study the microenvironment of PDAC since these devices allow imaging of cell–cell interactions, such as tumor–endothelial and tumor–immune cell interactions, as well as cell morphological changes, by applying different types of fluorescence microscopy techniques [4,74,75,76,77,78]. In a recent work by Hye-ran Moon et al., a microfluidic pancreatic tumor model was developed, recapitulating the heterogeneous driver mutations of human PCs by using PDAC cells derived from genetically engineered mouse models (KPC with *Kras* and *Trp53* mutations, and KIC with *Kras* mutation and *Cdkn2a* deletion) in order to mimic the intra-tumoral heterogeneity (Figure 4a) [77]. The model was successfully used to study interaction mechanisms between heterogeneous cancer cell subpopulations exposed to anti-cancer drugs and associated drug resistance. By means of fluorescence microscopy, the authors observed significant morphological changes in the epithelial phenotype KIC (eKIC) cells co-cultured with the mesenchymal phenotype KIC (mKIC) cells at the level of enhanced gemcitabine resistance in the co-culture models (Figure 4b), suggesting that interactions between these two cancer cell types induced multiple changes of the eKIC cells including loss of epithelial characteristics, most likely causing increased resistance to gemcitabine. By means of confocal immunofluorescence, the authors assessed the changes in E-cadherin (E-cad) expression and observed that when co-culturing cells, E-cad expression in eKIC was significantly reduced, implying that the interactions between heterogeneous cancer cells may induce the phenotype transition of epithelial cancer cell types. In a different approach, Nguyen and colleagues reported a new organotypic PDAC-on-a-chip model that mimics vascular invasion and tumor–blood vessel interactions (Figure 4c–f) [79]. The microfluidic device is composed of two hollow cylindrical channels embedded within a 3D collagen matrix. One channel is seeded with endothelial cells to form a perfusable biomimetic blood vessel, while the other channel is seeded with PC cells to form a pancreatic cancer duct. To study the interactions of PDAC cells with the blood vessels, the authors performed a screening experiment wherein different chemotactic agents were introduced into the biomimetic blood vessel and found that a gradient of fetal bovine serum most efficiently stimulated the invasion of PC cells into the collagen matrix. By means of confocal microscopy, the authors were able to record the ablation of blood vessel by cancer cells—notably, they observed that once in contact with the biomimetic blood vessel, the PDAC cells wrapped around the blood vessel and spread along the length of the blood vessel before invading into the vessel itself, leaving behind tumor-lined and tumor-filled luminal structures (Figure 4c-f). The infiltration of immunosuppressive cells is critical in the generation and maintenance of an immunosuppressive environment in PDAC, thus contributing to the failure of current available therapeutic approaches. In this regard, cell–cell interaction studies have revealed an important interplay between tumor-associated macrophages and regulatory T cells (Tregs). By using light sheet fluorescent microscopy, Siret C. et al. were able to show a direct interaction between myeloid-derived suppressor cells (MDSCs) and Tregs cells in a 3D PDAC tumor context [80]. These findings were also corroborated by the use of the transwell system, which demonstrated that cell-to-cell interactions are required for Treg cell proliferation and development induced by MDSCs. However, Treg cells were also shown to modulate proliferation and survival of MDSCs. Remarkably, by coupling imaging approaches and functional assays, the authors were able to show that physical interactions between cells contribute to the establishment of an immunosuppressive environment in PDAC. 

## 5. The End of Perpetual Chemotherapy: Nanoparticles (NPs) for Cell Targeting 

**Figure 5:**
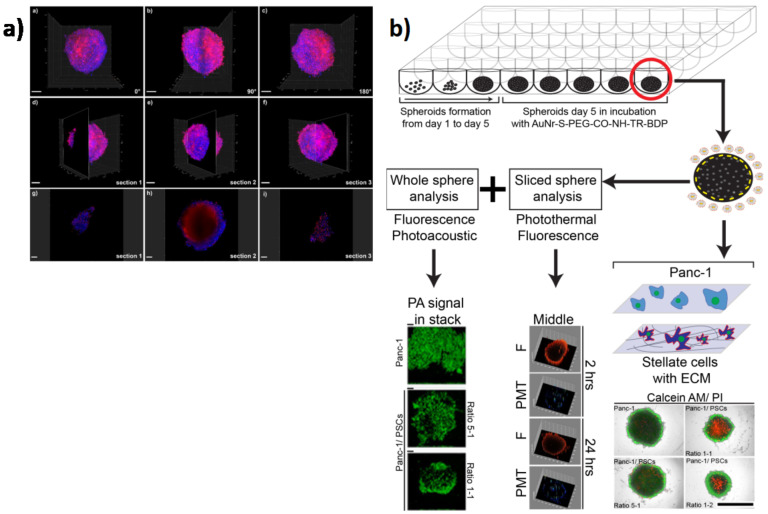
Nanoparticle applications in 3D in vitro models of PC. (**a**) 3D tomography with light sheet fluorescence microscopy of large hetero-type multicellular tumor spheroids after incubation with doxorubicin (Dox). Overlays of blue (nuclei, Hoechst 33342 staining) and red (Dox) channels. (**a**) 0° rotation, (**b**) 90° rotation, and (**c**) 180° rotation. Localization of (**d**) section 1 (113μm depth), (**e**) section 2 (403μm depth), and (**f**) section 3 (861μm depth) in relation to the entire spheroid. Scale bars: 200µm. Images taken at (**g**) section 1, (**h**) section 2, and (**i**) section 3. Scale bars: 100µm (adapted from [115]). (**b**) Schematic of the experimental approach: spheroid formation (day 1 to day 5) in ultra-low-attachment 96-well plate, functionalized gold nanodor (AuNR) incubation, followed by characterization of the whole sphere and a section of it (adapted from [116]).

For decades, gemcitabine (Gem)-based therapy has been the first-line treatment for patients with PC with an overall improvement in survival of only 6 months, where surgery is not an option [81]. Most recently, FOLFIRINOX (consisting of oxaliplatin, irinotecan, fluorouracil, and leucovorin) has achieved a slight increase in patient survival compared with Gem alone; however, it is associated with numerous side effects, including diarrhea, anemia, and increased risk of infections [82]. The presence of a dense desmoplastic stroma within PDAC tumors is the principle cause of chemotherapy failure, since it leads to an increased interstitial fluid pressure (IFP), which implies hypovascularity, reduced tumor perfusion, and the generation of a hypoxic environment [83]. Therefore, the stroma acts as a physical and biological barrier that inhibits drug delivery, thus contributing to therapy resistance [84]. In this scenario, nanomedicine has changed the world of cancer therapy. Nano-sized vehicles encapsulating drugs exhibit greater cellular uptake and prolonged circulation compared to classical chemotherapy. They possess the capability to overcome biological barriers, protect drugs from degradation, and foster their accumulation at the target site, and they are associated with reduced side effects [85,86]. Nanocarriers have been developed on the basis of natural (e.g., lipid, polyethylene glycol, poly(lactide-o-glycolic) acid, and chitosan) polymers or inorganic nanoparticles (e.g., gold, magnetic, mesoporous silica, and quantum dots) [87,88]. The first two FDA-approved nanomolecules for PDAC treatment were Abraxane, an albumin-bound paclitaxel-containing nanoparticle (NP), and Onivyde, an irinotecan liposome-based injection, which induced an increase in patient survival of 2-4 months compared to frontline treatment [89,90]. At the same time, strategies aimed at the disruption of stromal barriers to improve drug delivery have been explored with slight success [91]. Caution must be taken at the level of eliminating the stroma barrier (i.e., CAFs), as was exemplified by the failure of the Infinity Pharmaceuticals trial using IPI-926-03, a drug that depletes tumor-associated stromal tissue by inhibition of the HH cellular signaling pathway. Specifically, the trial was halted after patients in the gemcitabine + IPI-926 arm showed reduced overall survival compared to gemcitabine alone. Nonetheless, since this trial, new studies using, for example, vismodegib (formerly known as GDC-0449) have confirmed therapeutic effectiveness and response and have been evaluated in multiple clinical trials with other tumor entities (ClinicalTrials.gov Identifier: NCT02465060). The majority of nanoparticles have been designed for a controlled cargo release in response to distinct endogenous/exogenous stimuli such as pH level [92], temperature [93], redox reactions [94], enzyme activity [95], magnetic/electric field [96], mechanical force, and ultrasound/light irradiation [97]. Most recently, a new class of theranostic NPs have emerged, which enable simultaneous diagnosis, targeted delivery, and monitoring of therapy response [98,99]. Rosenberger et al. co-encapsulated the magnetic iron oxide NPs (IONPs) and the fluorescein isothiocyanate (FITC) dye within hyaluronic acid (HA)-functionalized NPs. This system takes advantage of the specific recognition of HA by the CD44 receptor (highly expressed on PDAC cells) and combines magnetic resonance with fluorescence imaging [100]. PC is considered a “cold tumor” with limited antigenic expression, making this tumor less likely to respond to immunotherapy. Several efforts have been evaluated, such as using PDT, chemotherapy, and other strategies to make PC more immunogenic [101]. In the last decades, tumor immunotherapy has emerged as a promising alternative to chemotherapy, but the inherent immune-suppressive pancreatic TME represents a challenge for its success. The use of specific NPs to activate the immune system within the TME is evolving as an efficient strategy to improve immunotherapy efficacy [102,103]. In this regard, one of the most promising approaches consists in targeting the interaction between the programmed death ligand 1 (PD-L1) with its receptor (PD-1), expressed by T lymphocytes. The PD-L1 protein is present on the surface of several cancer cells and its binding with PD-1 inhibits the activation of T cells, thus suppressing the immune response [104]. Mia and colleagues formulated a lipid-based (LPD) nanocarrier embedding a plasmid encoding for a fusion protein (called trap), specifically binding CXCL12 and PD-L1 for targeting PC [105]. The LPD nanocarriers were injected into mice bearing orthotopic PC tumors and were found to bind CXCL12, thus facilitating CD3^+^T-cell infiltration into the tumor. This therapy significantly reduced metastasis through the stimulation of the immunosuppressive TME. It is known that indoleamine 2,3-dioxygenase (IDO) enzyme, frequently overexpressed in tumors, contributes to the immunosuppressive microenvironment of cancer cells by suppressing the T cell proliferation through the depletion of tryptophan [106]. Lu and colleagues induced the suppression of the IDO pathway with the induction of immunogenic cell death (ICD) by using mesoporous silica nanoparticles (MSNPs) encapsulating the IDO inhibitor together with oxaliplatin. This system allowed for the recruitment of cytotoxic T lymphocytes and the downregulation of Foxp3+ T cells in vivo, leading to the reduction of tumor volume [107]. In addition to drug release, NPs can also encapsulate small RNA molecules, alone or with drugs, to silence cancer-causing genes and suppress tumor growth [108]. All these different approaches can be improved with the surface-functionalization of NPs with specific tumor-targeting ligands or antibodies for site-specific drug delivery [109]. NPs can have precise light interaction characteristics, making them suitable for several detection techniques, such as magnetic resonance imaging [110], Raman/SERS (Surface Enhanced Raman Spectroscopy) spectroscopy [111,112], two-photon microscopy [113], and photoacoustic analysis [114]. However, few of these techniques have been applied in 3D cancer models. Lazzari and colleagues tracked a doxorubicin-loaded polymeric NP diffusion in a complex PC 3D model (composed by cancer, endothelial, and fibroblast cells) using confocal laser scanning microscopy (CLSM). Their results clearly showed that CLSM was not suitable to accurately monitor the diffusion of small molecules such as doxorubicin due to the progressive loss of their fluorescence signal [115] (Figure 5a). Because complex 3D cancer models are designed to resemble the in vivo environment with low pH, the drugs/fluorescent dyes could detach from the surface of NPs before entering cells. Recently, Darrigues et al. generated spheroids by co-culturing PC cells and PSCs in various ratios to mimic different tumor–stromal compositions and to explore NP penetration. The authors used fluorescence live imaging, photothermal analysis, and photoacoustic analysis to observe nanoparticle performance in the spheroids, finding that the nanorods enabled multi-imaging detection even when fluorescence tracking was not possible [116] (Figure 5b). Despite the possible drawbacks, 3D models are still superb tools for understanding how multi-functional NPs interact with the in vivo environment.

## 6. Conclusions

**Figure 6:**
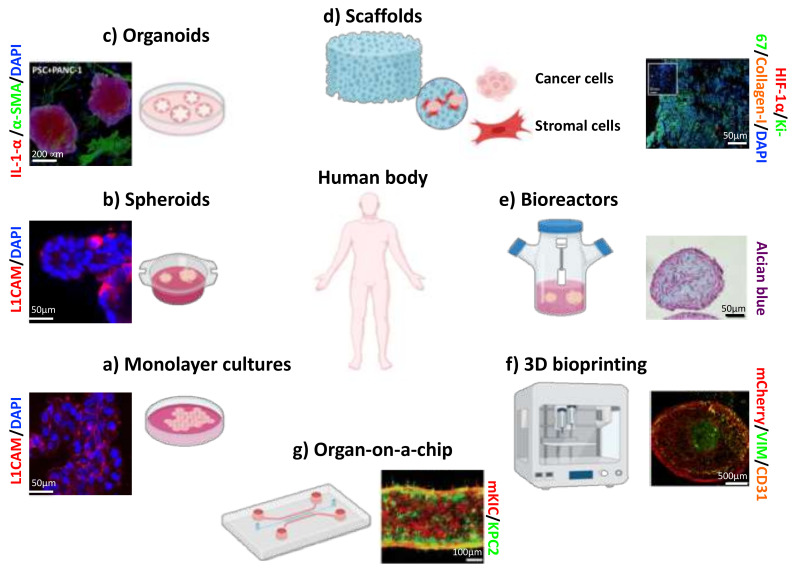
The evolving strategies for culturing and studying PC cells from human tissues. Representative immunofluorescence images of L3.6pl PDAC cells grown as monolayers (**a**) or spheroids (**b**) (adapted from [34]). (**c**) 3D confocal image of organoids generated by co-culturing PSCs (green) with PANC-1 (red) cells (adapted from [53]). (**d**) Fluorescent image of PANC-1 cells co-cultured with stromal cells on collagen-I scaffold (adapted from [42]). (**e**) Detection of glycosaminoglycans by Alcian blue staining in a homotypic 3D bio-reactor culture of human CAFs generated by bioengineered tumoral microtissues (adapted from [62]). (**f**) Representative immunofluorescence image of 3D bioprinted tissues for PDAC (green), PSC (red), and endothelial (yellow) cells (adapted from [68]). (**g**) IF image of the invasion of a heterogeneous co-culture of PDAC cells (adapted from [75]).

It is widely recognized that spatial and temporal cell–cell communications in the microenvironment contributes to PC initiation and progression. To study the complex cell–cell interactions, researchers must implement innovative experimental and analytical strategies. Recently, the development of in vitro 3D patient-derived cancer models has emerged as a revolutionary approach for effective in vitro anticancer drug screening. Furthermore, next-generation platforms of anticancer drugs, including nanoparticle-based delivery agents, will focus on the malignant phenotype as a whole and not just on cell proliferation. Thus, 3D cancer models provide an exceptional platform for studying critical cancer events not obtainable with other models. For instance, xenograft models resemble the original tissue but are expensive, time-consuming, and not suitable for high-throughput drug screening. Another promising avenue is the creation of tumor-on-a-chip platforms. This technology allows for reconstructing the cancer complexity with a dynamic physical microenvironment and, by linking different physiological modules, including vasculature, can be used to investigate the interactions between cancer and other organs. Certainly, all these models have several advantages and disadvantages, and there is no single method that satisfies all research needs. For this reason, further studies are required for the development of innovative in vitro pre-clinical models that can better overcome the limitations of these systems, leading to an improvement in personalized medicine (Figure 6 and Table 1). The use of in vitro 3D tumor models coupled with advanced high throughput and automated imaging techniques remains a promising direction for accelerating translation of these 3D cancer cultures into clinically relevant models for personalized medicine in the treatment of PC. 

## Figures and Tables

**Table 1:** Advantages and disadvantages of in vitro 3D culture models.

Technique	Advantages	Disadvantages
**Monolayer cultures**	Cost effective	Shape changed from original tissue (polarization lost)
a	Easy-to-use protocol	Lack vasculature
	Scalable to different plate formats	Reduces cell-to-cell interactions
	Compliant with high-throughput screening (HTS)	Less biologically relevant models
	High reproducibility	
	Formed from primary cells and cell lines	
**Spheroids**	Easy-to-use protocol	Simple architectures
	Scalable to different plate formats	Cannot control uniformity (size, composition)
	Compliant with high-throughput screening (HTS)	Not all cell lines form spheroids
	Formed from primary cells and cell lines	Agglomeration
	Long-term culture	Necrotic cores
	Allows co-cultures	Lack vasculature
**Organoids**	Patient-specific	Costly
	Scalable to different plate formats	Not easy-to-use protocol
	Formed from primary cells	Cannot control uniformity (size, composition)
	Allows co-cultures	Less amenable to HTS
	In vivo-like complexity	May lack key cell types
	In vivo-like architecture	Lack vasculature
	Amenable for tissue engineering and transplantation	Require validation to identify outgrowth of unwanted cells
		Requires access to human samples
**Scaffolds**	Scalable to different plate formats	Costly
	Compliant with high-throughput screening (HTS)	Not easy-to-use protocol
	High reproducibility	Simple architectures
	Formed from primary cells and cell lines	Batch-to-batch variability of natural matrixes
	Long term culture	Might require complex cell retrieval/imaging methods
	Allows co-cultures	
	In vivo-like complexity	
	In vivo-like architecture	
	Amenable for tissue engineering and transplantation	
	Naturally-derived ECM components of synthetic polymers	
	Resemble mechanical forces in tumors	
	Versatile	
	Tunable composition	
**Bioreactors**	High density cell expansion	Costly
	Controllable culture parameters	Requires optimization of cell parameters and biomaterial inclusion
		Hydrodynamic shear stress
**Organ-on-a-chip**	Compliant with high-throughput screening (HTS)	Costly
	High reproducibility	Requires special equipment
	Formed from primary cells and cell lines	Difficult to scale up
	Allow co-cultures	
	In vivo-like complexity	
	In vivo-like architecture	
	Controllable culture parameters	
	Vascularized	
**3D Bioprinting**	High-throughput production	Costly
	High reproducibility	Requires special equipment
	Formed from primary cells and cell lines	Challenges with cells/materials
	Allow co-cultures	Issues with tissue maturation
	In vivo-like complexity	Needs optimization
	In vivo-like architecture	
	Controllable culture parameters	
	Vascularized	
